# Determinant factors for loss to follow-up in drug-resistant tuberculosis patients: the importance of psycho-social and economic aspects

**DOI:** 10.1186/s12890-021-01735-9

**Published:** 2021-11-10

**Authors:** Soedarsono Soedarsono, Ni Made Mertaniasih, Tutik Kusmiati, Ariani Permatasari, Ni Njoman Juliasih, Cholichul Hadi, Ilham Nur Alfian

**Affiliations:** 1 Department of Pulmonology and Respiratory Medicine, Faculty of Medicine, Universitas Airlangga, Surabaya, Indonesia; 2 Department of Clinical Microbiology, Faculty of Medicine, Universitas Airlangga, Surabaya, Indonesia; 3 Laboratory of Tuberculosis, Institute of Tropical Disease, Universitas Airlangga, Surabaya, Indonesia; 4 Department of Psychology, Faculty of Psychology, Universitas Airlangga, Surabaya, Indonesia; 5 Tuberculosis Study Group, Universitas Airlangga, Surabaya, Indonesia

**Keywords:** Drug-resistant tuberculosis, Loss to follow-up, Psycho-social support, Economic support

## Abstract

**Background:**

Drug-resistant tuberculosis (DR-TB) is the barrier for global TB elimination efforts with a lower treatment success rate. Loss to follow-up (LTFU) in DR-TB is a serious problem, causes mortality and morbidity for patients, and leads to wide spreading of DR-TB to their family and the wider community, as well as wasting health resources. Prevention and management of LTFU is crucial to reduce mortality, prevent further spread of DR-TB, and inhibit the development and transmission of more extensively drug-resistant strains of bacteria. A study about the factors associated with loss to follow-up is needed to develop appropriate strategies to prevent DR-TB patients become loss to follow-up. This study was conducted to identify the factors correlated with loss to follow-up in DR-TB patients, using questionnaires from the point of view of patients.

**Methods:**

An observational study with a cross-sectional design was conducted. Study subjects were all DR-TB patients who have declared as treatment success and loss to follow-up from DR-TB treatment. A structured questionnaire was used to collect information by interviewing the subjects as respondents. Obtained data were analyzed potential factors correlated with loss to follow-up in DR-TB patients.

**Results:**

A total of 280 subjects were included in this study. Sex, working status, income, and body mass index showed a significant difference between treatment success and loss to follow-up DR-TB patients with *p*-value of 0.013, 0.010, 0.007, and 0.006, respectively. In regression analysis, factors correlated with increased LTFU were negative attitude towards treatment (OR = 1.2; 95% CI = 1.1–1.3), limitation of social support (OR = 1.1; 95% CI = 1.0–1.2), dissatisfaction with health service (OR = 2.1; 95% CI = 1.5–3.0)), and limitation of economic status (OR = 1.1; 95% CI = 1.0–1.2)).

**Conclusions:**

Male patients, jobless, non-regular employee, lower income, and underweight BMI were found in higher proportion in LTFU patients. Negative attitude towards treatment, limitation of social support, dissatisfaction with health service, and limitation of economic status are factors correlated with increased LTFU in DR-TB patients. Non-compliance to treatment is complex, we suggest that the involvement and support from the combination of health ministry, labor and employment ministry, and social ministry may help to resolve the complex problems of LTFU in DR-TB patients.

## Introduction

Tuberculosis (TB) remains a global health problem caused 10 million people fell ill with TB and 1.2 million deaths. Drug-resistant TB (DR-TB) continues to be a public health threat. There were an estimated 465,000 DR-TB cases. Indonesia ranks 5th for high DR-TB burden with 24,000 DR-TB cases. DR-TB is the barrier for global TB elimination efforts with a global treatment success rate of 57% for DR-TB. Loss to follow-up (LTFU) is one of the main factors affecting low success rate of DR-TB treatment. In Indonesia, treatment success rate was below 50% due to high rates of LTFU (26%) [1].

Non-adherence to treatment is important concern for clinicians when managing infectious diseases, especially for the governments when making the public health policies as a strategy to end TB [1]. LTFU is a serious problem, causes mortality and morbidity for patients, and leads to wide spreading of DR-TB to their family and the wider community, as well as wasting health resources [3]. DR-TB requires treatment with second-line drugs, which have many more adverse effects than first-line anti-TB drugs. The treatment duration ranged from 9 to 11 months for shorter regimen and 18 to 24 months for longer regimen, depending on the treatment regimen. Therefore, compliance and motivation of patients during treatment should be maintained to complete treatment and achieve cure [1, 4].

Previous studies reported adverse drug reactions (ADRs) as the most important factors associated with LTFU, as DR-TB treatment was longer and greater incidence of ADRs compared to drug-susceptible TB [5–9]. A long duration, greater incidence of ADRs, and unfavorable social conditions (unemployment and homelessness) are considered to be correlated with LTFU in DR-TB treatment [5, 6]. The adverse effects of treatment and history of previous DR-TB treatment were also associated with non-adherence to anti-TB treatment [7–9]. However, non-treatment factors such as psycho-social and economic may also played role in LTFU. The problem of LTFU in DR-TB treatment also may be more complex in Indonesia, as one of the countries with a high DR-TB burden.

Prevention and management of LTFU is crucial [1]. Knowing the factors associated with LTFU could be used to develop appropriate strategies to prevent DR-TB patients become LTFU. This was important to reduce mortality, prevent further spread of DR-TB, and inhibit the development and transmission of more extensively drug-resistant strains of bacteria [10]. This present study was conducted to identify the factors correlated with LTFU in DR-TB patients, using questionnaires in the point of view of patients in aspects of treatment, psycho-social and economic.

## Methods

This was an observational study with a cross-sectional design. The samples were all DR-TB patients who have been declared as treatment success and LTFU from DR-TB treatment and received long regimen with injectable drug from 2017 to March 2021. Treatment success was defined as the sum of cured and treatment completed. Cured was defined as treatment completed as recommended by the national policy without evidence of failure and three or more consecutive cultures taken at least 30 days apart are negative after the intensive phase. Treatment completed was defined as treatment completed as recommended by the national policy without evidence of failure but no record that three or more consecutive cultures taken at least 30 days apart are negative after the intensive phase. LTFU was defined as a patient whose treatment was interrupted for 2 consecutive months or more [11]. Respondents included DR-TB patients who come from residents in the area of Surabaya and surrounding areas of East Java, Indonesia.

A structured questionnaire was used to collect information by interviewing the subjects as respondents who have signed the informed consent. The questionnaire was developed from any validated and previously published articles, and some questions were added from the experiences of clinicians when providing medical services and hearing the complaints of DR-TB patients. The questionnaire was administered when the treatment outcomes of DR-TB patients have been reported. The questionnaire was administered to DR-TB patients in a home visit by peer educators or patients’ supporters.

The questionnaire contained four aspects: negative attitude towards treatment, limitation of social support, health service, and limitation of economic status. Negative attitude towards treatment consisted of 30 questions, limitation of social support with 7 questions, dissatisfaction of health service with 1 question, and limitation of economic status consisted of 3 questions. A 2-point (yes and no) was used for negative attitude towards treatment. Every patient who answered yes (has a negative attitude towards treatment) for each question has a score of 1 (maximum score: 30), while every patient who answered no (has no negative attitude towards treatment) for each question has a score of 0 (minimum score: 0). While a 5-point scale (strongly disagree, disagree, neutral, agree, and strongly agree) was used for limitation of social support, dissatisfaction with health service, and limitation of economic status. Each question has a score of 1 for strongly disagree, 2 for disagree, 3 for neutral, 4 for agree, and 5 for strongly agree.

Negative attitude towards treatment in the questionnaire is the negative attitude and perception of subjects about DR-TB treatment. Questionnaire of limitation of social support described support from family, friends, co-workers, and other people when the subjects were diagnosed and treated for DR-TB. Questionnaire of health service described the view of subjects about the health services by the health-care worker in providing DR-TB treatment. Questionnaire of limitation of economic status comprised the questions of economic conditions while being treated with DR-TB treatment, including job, income, transport fee, and enablers from the government. Table 1 below is themes of questions in the questionnaire.

**Table 1:** Variables and themes of questions in the questionnaire

Variables	Themes of questions
Negative attitude towards treatment	Lack of awareness
	Myths and misbeliefs regarding disease
	Adverse drug and treatment effects
	Duration and schedule of medication conflicting with daily activities
Limitation of social support	Stigma and discrimination
	Lack of family and social support
Dissatisfaction with health service	Behavior of service provider
Limitation of economic status	Conflicting timing of job and treatment
	Unemployment and financial constraints
	Late of enablers payment from the government

Completed questionnaires are input by the research assistant and double checked by the investigators. Data was entered and analyzed using SPSS 21.0 (SPSS 21.0 by IBM Corporation, New York, United States) for all statistical analyses. Socio-demographic data were summarized as frequencies & percentages and were analyzed for significance test using Chi-square test. Logistic regression analysis was performed to determine potential factors correlated with LTFU in DR-TB patients. A *p* < 0.05 was considered statistically significant. This study was approved by the ethics committee with ethical clearance number 103/EC/KEPK/FKUA/2021.

## Results

Of the 350 DR-TB patients with treatment outcomes of treatment success and LTFU, a total of 280 subjects agreed to be interviewed and included in this study. 280 subjects consisted of 115 treatment success and 165 LTFU. Sex, working status, income, and body mass index showed significant differences between treatment success and loss to follow-up DR-TB patients with *p* = 0.013, 0.010, 0.007, and 0.006, respectively. While age, education level, marital status, and family history of TB disease between treatment success and LTFU DR-TB patients showed no significant difference. Socio-demographic of study subjects are presented in Table 2.

**Table 2:** Socio-demographic of study subjects

Variables	Treatment success	LTFU	Total (n = 280)	*P*-value
	N (%) or mean ± SD or median (IQR, 1st–3rd quartile)			
*Sex*				0.013
Male	65 (35.7%)	117 (64.3%)	182	
Female	50 (51%)	48 (49%)	98	
*Age*				0.367
	47.5 ± 11.4	44.4 ± 12.1	45.7 ± 11.9	
	(19–73)*	(16–75)*	(16–75)*	
*Education level*				0.635
Elementary school	29 (40.3%)	43 (59.7%)	72	
Junior high school	21 (38.9%)	33 (61.1%)	54	
Senior high school	55 (40.4%)	81 (59.6%)	136	
Diploma and above	10 (55.6%)	8 (44.4%)	18	
*Working status*				0.010
Jobless	55 (33.7%)	108 (66.3%)	163	
Non-regular Employee	35 (48.6%)	37 (51.4%)	72	
Regular employee	25 (55.6%)	20 (44.4%)	45	
*Income*				0.007
< 1 million rupiah	61 (34.7%)	115 (65.3%)	176	
> 1–3 million rupiah	37 (48.1%)	40 (51.9%)	77	
> 3 million rupiah	17 (63%)	10 (37%)	27	
*Marital status*				0.140
Single	11 (27.5%)	29 (72.5%)	40	
Married	89 (42.6%)	120 (57.4%)	209	
Divorced	15 (48.4%)	16 (51.6%)	31	
*Family history of TB disease*				0.148
Yes	9 (29%)	22 (71%)	31	
No	106 (42.6%)	143 (57.4%)	249	
*Body mass index*				0.006
Underweight	22 (30.6%)	50 (69.4%)	72	
Normal	72 (41.1%)	103 (58.9%)	175	
Overweight and obese	21 (63.7%)	12 (36.3%)	33	
Negative attitude towards treatment	14 (10–16)**	20 (14–22)**	280	< 0.001
Limitation of social support	15 (11–16)**	19 (16–23)**	280	< 0.001
Dissatisfaction with health service	2 (1–2)**	4 (2–4)**	280	< 0.001
Limitation of economic status	7 (5–9)**	6 (5–11)**	280	0.310

*Mean ± standard deviation (minimum–maximum)

**Median (IQR, 1st–3rd quartile)

Table 2 above also showed that LTFU patients have a significantly higher mean value of negative attitude towards treatment, compared to treatment success patients, with median (IQR) of 20 (14–22) versus 14 (10–16), *p* < 0.001. Limitation of social support was also found higher significantly in LTFU patients than in treatment success patients with median (IQR) of 19 (16–23) versus 15 (11–16), *p* < 0.001. LTFU patients also have a higher dissatisfaction with health service than those treatment success patients with median (IQR) of 4 (2–4) versus 2 (1–2), *p* < 0.001. While limitation of economic status in LTFU and treatment success patients have median (IQR) of 6 (5–11) versus 7 (5–9), *p* = 0.310.

Regression analysis in Table 3 showed that factors increased of being LTFU were negative attitude towards treatment (OR 1.2, 95% CI 1.1–1.3), limitation of social support (OR 1.1, 95% CI 1.0–1.2), health service (OR 2.1, 95% CI 1.5–3.0), and limitation of economic status (OR 1.1, 95% CI 1.0–1.2). This result also showed that although limitation in economic status was not significantly higher in LTFU patients, it has a significant impact on the prevalence of LTFU when analyzed in a logistic regression.

**Table 3:** Binary logistic regression analysis between complaints of patients and LTFU

Variables	OR	(95% CI)
Negative attitude towards treatment	1.2	(1.1–1.3)
Limitation of social support	1.1	(1.0–1.2)
Dissatisfaction with health service	2.1	(1.5–3.0)
Limitation of economic status	1.1	(1.0–1.2)

## Discussion

Socio-demographic of study subjects in Table 2 showed that sex, working status, income, and BMI between treatment success and LTFU patients were significantly different (*p*-value of 0.013, 0.010, 0.007, and 0.006, respectively). While age, education level, marital status, and family history of TB between treatment success and LTFU patients were not significant differences with *p* = 0.367, 0.635, 0.140, and 0.148, respectively. LTFU was found higher in males than females (64.3% vs. 49%, *p* = 0.013). A previous study reported that older age and male sex were risk factors for LTFU, whereas patients with higher initial body weight were less likely to be LTFU [12]. A significant association of LTFU with occupation, marital status, and socio-economic status in newly diagnosed pulmonary TB and extra pulmonary TB patients was also reported in a multi stratified study in India [3].

Loss to follow-up patients in our study were found higher in males, compared to females (64.3% vs. 49%, *p* = 0.013). Working status between treatment success and LTFU was also found significantly different (*p* = 0.010). A high rate of LTFU was found in patients who were unemployed and non-regular employees (66.3% and 51.4%). Male sex and working status affected LTFU probably because male patients often work to provide the needs of their families, while patients of regular employees have a lower rate of LTFU (44.4%) perhaps because they already have permanent jobs and no need to be worried when dividing their times between working and taking DR-TB treatment. Another study also reported that most of non-adherents were males patients, most of them were day-laborers and main earning members [13]. Going to a healthcare facility for DR-TB treatment for an employed patient means an absent time from work, and it may pose huge problems, especially for non-regular employees. Working and treatment may also put them in a stress condition, that as soon as they begin to feel better, they will choose to return to work to continue to earn for their families. While female patients, especially who were housewife may have more available time to take their drugs on proper time [3].

Patients with education levels of elementary school, junior high school, and senior high school have higher rates of LTFU, compared to patients with education levels of diploma and above, but the statistical analysis showed no significant difference (*p* = 0.635). Patients with education level of diploma and above have a lower rate of LTFU (44.4%), although it was not significant statistically. A study in China in Ethiopia found that anti-TB treatment non-adherence was associated with poor TB knowledge [14, 15]. In this study, the lower rate of LTFU in patients with higher education levels may be due to higher awareness and better knowledge of their disease, thus increase their compliance for treatment. A higher education level affected the way of thinking, including the ability to overcome problems [16], associated with better adherence to treatment since it increases awareness of the disease [17, 18].

Our study found that income between treatment success and LTFU patients was significantly different (*p* = 0.007). Most of subjects in this study have income below 1 million rupiah (rate of 65.3%), showing poor condition. Cost of transport and other needs during treatment are also problems for patients who are in poor condition, and LTFU became their final option [3]. The correlation between poverty and LTFU could be reduced by a strategy in programs, the supply of financial incentives may improve the adherence to treatment [17].

Using structured questionnaires, logistic regression analysis found that negative attitude towards treatment (OR 1.2, 95% CI 1.1–1.3), limitation of social support (OR 1.1, 95% CI 1.0–1.2), dissatisfaction with health service (OR 2.1, 95% CI 1.5–3.0), and limitation of economic status (OR 1.1, 95% CI 1.0–1.2) were significantly positively correlated with LTFU from treatment (Table 3). Our findings suggested that a strategy to improve treatment adherence needs to combine the aspect of psychological, social, health service, and economic support.

The aspect of negative attitude towards treatment comprised lack of awareness, myths and misbeliefs regarding the disease, adverse drug and treatment effects, duration and schedule of medication conflicting with daily activities. Education and counseling for DR-TB patients are very important to break the myths and misbeliefs among patients regarding disease, also to inform the patients about the benefits of medication over the adverse effects. A previous study also reported drug side effects and conflicts with the timing of treatment services as the barriers to treatment adherence [19]. Another study found that poor adherence to DR-TB treatment is associated with negative side effects from the treatment, busy work schedules, and financial difficulties [8]. In patients with multidrug-resistant tuberculosis (MDR-TB), both patient and regimen were related factors associated with LTFU [20]. A case–control study in Tajikistan reported that patients who have been previously treated need extra care to ensure treatment completion [21].

Treatment adherence is influenced by many factors, including socio-economic factors and drug toxicity, perceived health benefits, and subjective experience of illness [3]. Independent factors associated with LTFU included patients’ higher self-rating of the severity of adverse drug reaction, while protective factors included receiving any type of assistance from the TB program, better TB knowledge, and higher levels of trust in and support from physicians and nurses [22]. Treatment outcomes were mainly affected by patient individual factors [23].

Limitation of social support in this study included stigma, discrimination, and lack of family and social support had a significant correlation with LTFU (OR 1.1, 95% CI 1.0–1.2) (Table 3). Non-adherence to treatment correlated with lack of provider support and social stigma. Resolving medical problems like adverse drug effects, motivational counseling, flexible timings for health-care services, social, family support for patients & improving awareness about disease were required to be enhanced [19]. In certain patients, motivation to continue treatment decreases over time, and when they feel their conditions have improved, they may LTFU from treatment [3]. Patients will need support to overcome the hardships associated with TB and its treatment, including daily adherence, adverse drug reactions, indirect costs, and stigma [4]. Counseling based on behavioral activation theory, information/education materials, and group interactions with other patients showed acceptable to patients to resolve their depression during treatment, suggested the need for counselors in TB clinics [24].

Dissatisfaction with health service (from the physicians and nurses) such as poor communication between patients and healthcare workers was associated with LTFU [25]. A good communication between health care providers, patients, and their families, and strong social support networks could reduce the stigma [17]. Economic status, including conflicting timing of job and treatment, financial constraints, and late payment of enablers from government also play role in LTFU in our study. Although medicines are provided free, but family liabilities and burden of losing income from work were possible to cause LTFU [3].

Enablers for transportation may minimize the financial barrier to adherence. However, the delay of payment is a problem. The amount of assistance from enablers is limited and transportation cost may exceeds the financial ability of patients, and loss of income when undergoing treatment at health-care facility which is not open for full-day [10]. The World Health Organization (WHO) also reported that DR-TB patients and their households faced higher catastrophic costs than drug-susceptible TB (DS-TB) patients, including the combined cost of transportation, food, nutritional supplements, and other non-medical expenditures [1]. Improving treatment adherence is needed, including providing material support (e.g. food, financial incentives, and reimbursement of transport fees) and psychological support [4]. Factors influencing patient adherence to TB treatment are factors of patient-centered, social, economic, health system, therapy, lifestyle, and geographic access [26]. Psycho-emotional and socio-economic interventions provided to TB patients showed beneficial effects on TB treatment outcomes [27].

Certain LTFU patients may lose their jobs due to undergoing treatment at a healthcare facility. Economic factors such as employment status and the need to borrow money when seeking treatment may also influence LTFU [10]. Helping patients to achieve full adherence to TB medication is a complex problem as it is influenced by interplay between many factors. Healthcare managers, providers, and researchers need to consider and address multiple underlying factors when designing adherence interventions [26].

Loss to follow-up from DR-TB treatment is a barrier to cure and control the disease [28]. LTFU patients are a threat to the spread of DR-TB disease in the community. Identified factors correlated with LTFU can be used to make a strategy to resolve this urgent problem [29], it is also essential to prevent the community from primary DR-TB infection and to reduce further drug resistance developments [30]. Non-compliance to treatment is complex [3], the role and efforts from all parties are essential. The involvement and support from the combination of health ministry, labor and employment ministry, and social ministry may help to resolve the complex problems of LTFU in DR-TB patients.

## Strength and limitations

The strength of this study was that it was the first in this kind reported from Indonesia, especially when adverse drug reaction of DR-TB treatment was considered as the most important factor for LTFU. Thus, the authorities should evaluate psycho-social and economic factors of patients. The results of this study were also as a policy intake to strengthen the collaboration between health ministry, labor and employment ministry, and social ministry as a strategy to prevent LTFU in DR-TB patients. The limitation of this study was that the respondents were possible to have a tendency to report an answer in a way they deem more acceptable and more appropriate instead of their true thoughts and experiences.

## Conclusions

Male patients, unemployment, non-regular employee, lower income, and underweight BMI were found higher in LTFU patients. Negative attitude towards treatment, limitation of social support, dissatisfaction with health service, and limitation of economic status are factors correlated with increased LTFU in DR-TB patients. Non-compliance to treatment is complex, we suggest that the involvement and support from the combination of health ministry, labor and employment ministry, and social ministry may help to resolve the complex problems of LTFU in DR-TB patients.

## Data Availability

The datasets analyzed during the current study are available from the corresponding author on reasonable request.

## Abbreviations

ADRs: Adverse drug reactions
Anti-TB: Anti-tuberculosis
BMI: Body mass index
CI: Confidence interval
DR-TB: Drug-resistant tuberculosis
DS-TB: Drug-susceptible TB
LTFU: Loss to follow-up
OR: Odds ratio
TB: Tuberculosis
WHO: World Health Organization
